# Ascorbic acid improves parthenogenetic embryo development through TET proteins in mice

**DOI:** 10.1042/BSR20181730

**Published:** 2019-01-11

**Authors:** Wei Gao, Xianfeng Yu, Jindong Hao, Ling Wang, Minghui Qi, Liang Han, Chao Lin, Dongxu Wang

**Affiliations:** 1 Laboratory Animal Center, College of Animal Science, Jilin University, Changchun 130062, China; 2 Department of Emergency, First Hospital, Jilin University, Changchun 130031, Jilin, China

**Keywords:** Ascorbic acid, DMOG, Parthenogenetic embryo, TET proteins, 5hmC

## Abstract

The TET (Ten-Eleven Translocation) proteins catalyze the oxidation of 5mC (5-methylcytosine) to 5hmC (5-hydroxymethylcytosine) and play crucial roles in embryonic development. Ascorbic acid (Vc, Vitamin C) stimulates the expression of TET proteins, whereas DMOG (dimethyloxallyl glycine) inhibits TET expression. To investigate the role of TET1, TET2, and TET3 in PA (parthenogenetic) embryonic development, Vc and DMOG treatments were administered during early embryonic development. The results showed that Vc treatment increased the blastocyst rate (20.73 ± 0.46 compared with 26.57 ± 0.53%). By contrast, DMOG reduced the blastocyst rate (20.73 ± 0.46 compared with 11.18 ± 0.13%) in PA embryos. qRT-PCR (quantitative real-time PCR) and IF (immunofluorescence) staining results revealed that TET1, TET2, and TET3 expressions were significantly lower in PA embryos compared with normal fertilized (Con) embryos. Our results revealed that Vc stimulated the expression of TET proteins in PA embryos. However, treatment with DMOG significantly inhibited the expression of TET proteins. In addition, 5hmC was increased following treatment with Vc and suppressed by DMOG in PA embryos. Taken together, these results indicate that the expression of TET proteins plays crucial roles mediated by 5hmC in PA embryonic development.

## Introduction

The TET (Ten-Eleven Translocation) proteins are α-KGDDs (α-ketoglutarate- and Fe^2+^-dependent dioxygenases) that catalyze the oxidation of 5mC (5-methylcytosine) to 5hmC (5-hydroxymethylcytosine) during early mammalian embryogenesis [1]. Accumulating data indicate that TET1 functions in the erasure of paternal imprints in the female germline [2]. In addition, TET2 promotes hematopoietic stem cell self-renewal and myeloid transformation in the development to term in mice [3]. TET3 mediates epigenetic reprogramming in early embryos, and its conditional knockout causes an increased incidence of developmental failure and reproductive defects [4]. These lines of evidence indicate that TET proteins are associated with embryo development.

PA (parthenogenetic) embryos have been extensively used to study epigenetic profiles due to the lack of paternal gene expression [5,6]. Several epigenetic modifications, such as DNA methylation and histone modification, have been observed during the development of PA embryos [7]. In addition, down-regulated expression of TET1, TET2, and TET3 has been confirmed in PA embryos [8].

A recent report suggested that Vc (Vitamin C), as a cofactor for α-KGDDs, increases TET expression in human cells [9]. DMOG (dimethyloxallyl glycine) is a non-specific TET inhibitor that suppresses TET expression [10]. In the present study, Vc and DMOG were used to evaluate the role of TET proteins in PA embryonic development. The expression patterns of TET1, TET2, and TET3 were determined using qRT-PCR (quantitative real-time PCR) and IF (immunofluorescence) staining.

## Materials and methods

### Ethics statement

The experiments involving mice were carried out in accordance with the guidelines on animal care and use of animals in research approved by the Animal Care and Use Committee of Jilin University, Changchun, China (Grant No. 201706005).

### Production of normally fertilized and PA embryos

Female C57BL/6 mice (6–8 weeks old) were obtained from the School of Medical Science, Jilin University. For the superovulation test, female mice were superovulated by intraperitoneal injection of 10  IU of PMSG (pregnant mare serum gonadotropin; Merck Millipore) followed by intraperitoneal injection of 10  IU of hCG (human chorionic gonadotropin; Sigma) 48 h later. Subsequently, the female mice were individually mated with C57BL/6 males of proven fertility. The females were killed by cervical dislocation, and the oviducts were removed. Two-celled-stage embryos were collected into droplets of pre-equilibrated M2 medium (Sigma). The two-celled-stage embryos were then washed and cultured in M16 medium overlaid with mineral oil and incubated at 37°C in a humidified atmosphere of 5% CO_2_ in air until they developed to the blastula stage.

For the production of PA embryos, the oviducts of female mice were removed, and COCs (cumulus–oocyte complexes) were collected as unfertilized oocytes after PMSG and hCG injection. The cumulus was removed and collected by briefly exposing the MII oocytes to serum‐free medium containing hyaluronidase (Sigma). The oocytes were treated with a calcium ionophore (ionomycin calcium salt; Sigma) for 5 min. Parthenogenesis was activated after incubation of the unfertilized oocytes in M16 medium containing 6-DMAP (2 mmol/l, Sigma) for 4  h. Then, these unfertilized oocytes were transferred to fresh M16 medium. PA activation was confirmed by the presence of two pronuclei, which developed to the two-celled stage. These PA embryos (two-celled stage) were then transferred to fresh M16 medium and incubated until they reached the blastocyst stage.

### Drug treatment

Vc (100 nM, Sigma–Aldrich) and DMOG (600 nM, Sigma–Aldrich) were diluted in M16 medium. Two-celled-stage embryos were cultured in M16 medium with Vc or DMOG for 72 h. The blastocyst rates of Con and PA embryos were calculated after 72 h of culture.

### Gene expression analysis

Total RNA was extracted from each group of embryos (*n*=10) using the AllPrep DNA/RNA Micro Kit (QIAGEN, Germany) following the manufacturer’s instructions. cDNA was synthesized using the First-Strand cDNA Synthesis kit (Promega, U.S.A.). qRT-PCR was performed to determine TET1, TET2, and TET3 expressions using the BioEasy SYBR Green I Real-Time PCR Kit on a Bio-Rad iQ5 Multicolor Real-Time PCR Detection System (Bioer Technology, China). The primer sequences used in the present study are listed in Supplementary Table S1. PCR was performed by initial denaturation at 95°C for 3 min, followed by 40 cycles of denaturation at 95°C for 10 s, annealing at 60°C for 15 s, and extension at 72°C for 30 s. The 2^−ΔΔ*C*^_T_ method was used to determine relative gene expression. The experiments were performed at least in triplicate.

### IF staining

Briefly, the embryos were washed three times in PBS–PVA. Then, thinning of the zona pellucida was performed using Tyrode’s Solution (Jisskang, China). The embryos were fixed with 4% paraformaldehyde for 30 min at RT (room temperature). After fixation, the embryos were washed with PBS–PVA and permeabilized with PBS containing 0.2% Triton X-100 for 30 min. For 5mC and 5hmC staining, DNA was denatured using HCl (2 N) for 30 min at 37°C and subsequently treated with 0.1 M Tris/HCl (pH 8.0) for 15 min. Embryos were then incubated in PBS containing 1% BSA for 1 h. Next, the embryos were probed with different primary antibodies as follows: 5mC (1:100, Cell Signaling Technology), 5hmC (1:100, Cell Signaling Technology), TET1 (1:100, Abcam), TET2 (1:100, Abcam), and TET3 (1:100, Abcam) and incubated at 4°C overnight. The embryos were washed with PBS three times for 10 min each followed by incubation with Alexa Fluor 488– or 594–conjugated secondary (anti-mouse or anti-rabbit) antibodies for 1 h at RT. DNA was stained with 10 ng/ml Hoechst 33342 (Thermo Scientific) for 15–20 min. The embryos were washed thrice with PBS–PVA for 10 min each, air dried, and mounted on a coverslip and a glass slide using an antifade mounting medium (BOSTER, China). The embryos were imaged using confocal laser scanning microscopy. To evaluate the average fluorescence intensity in the embryos, image analysis software (ImageJ) was used [11].

### Statistical analysis

All data were analyzed using the Statistical Package for the Social Sciences (SPSS Inc., Chicago, IL, U.S.A.). Student’s *t* test was used to compare differences between quantitative variables for qRT-PCR and IF staining data. A *P*-value of <0.05 was considered statistically significant.

## Results

### The effect of Vc and DMOG on PA embryo development

In the present study, Vc and DMOG were applied to the culture of PA embryos. As shown in Table 1, the cleavage rate (86.37 ± 0.22 compared with 89.08 ± 0.79%) and blastocyst rate (20.73 ± 0.46 compared with 26.57 ± 0.53%) were improved in Vc-treated PA embryos. By contrast, the cleavage rate (86.37 ± 0.22 compared with 54 ± 0.17%) and blastocyst rate (20.73 ± 0.46 compared with 11.18 ± 0.13%) were reduced by DMOG treatment. These results indicated that Vc and DMOG have roles in PA embryonic development.

**Table 1:** The effect of Vc and DMOG on embryo development

	Total number	Two-celled rate (%)	Blastocyst rate (%)
Con	280	100 ± 0% (280)	84.61 ± 0.24% (238)
Con-Vc	314	100 ± 0% (314)	89.13 ± 0.3% (279)^1^
Con-DMOG	184	100 ± 0% (184)	65.92 ± 0.1% (121)^1^
PA	412	86.37 ± 0.22% (358)^1^	20.73 ± 0.46% (90)^1^
PA-Vc	286	89.08 ± 0.79% (252)^1,2^	26.57 ± 0.53% (74)^1,2^
PA-DMOG	672	54 ± 0.17% (363)^1,2^	11.18 ± 0.13% (72)^1,2^

Different superscripts (1, 2) denote significant differences (*P*<0.05). ^1^Indicated comparison with Con embryos. ^2^Indicated comparison with PA embryos.

### Analysis of global DNA methylation

**Figure 1:**
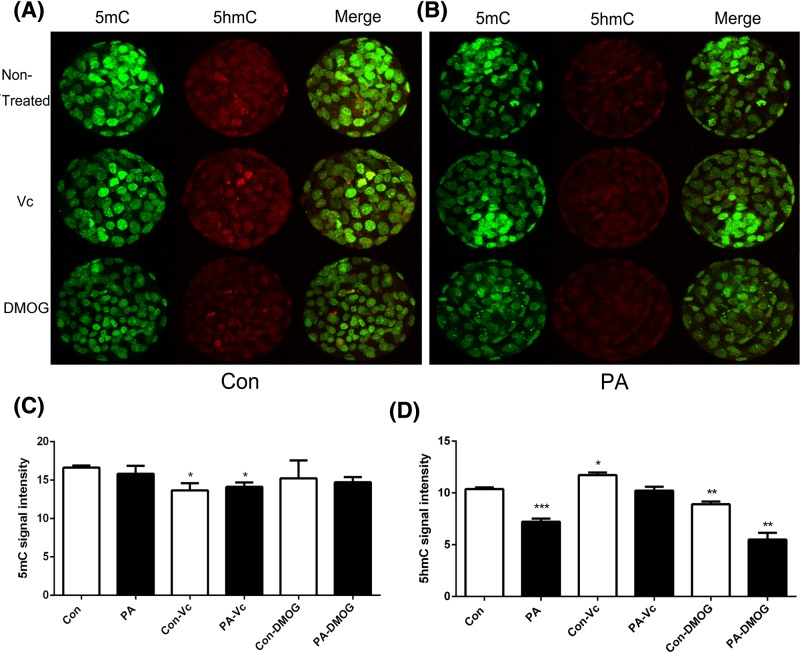
IF localization of 5mC and 5hmC The expression pattern of 5mC and 5hmC after Vc and VPA treated in Con (**A**) and PA (**B**) embryos. 5mC (**C**) and 5hmC (**D**) signal intensities were measured. Green, indicated 5mC. Red, indicated 5hmC. The data are represented as the mean ± S.E.M. (*n*=3). *P<0.05), **P<0.01, and ***P<0.005 indicate statistically significant differences.

To determine if the global DNA methylation level mediates embryo development, IF staining was performed to examine 5mC and 5hmC levels in blastocyst stage embryos. The results revealed lower 5hmC levels in PA embryos compared with the Con embryos group. Treatment with Vc significantly improved 5hmC levels in Con and PA embryos. DMOG suppressed 5mC and 5hmC in both Con and PA embryos (Figure 1). These results indicated that 5hmC was up-regulated by Vc and down-regulated by DMOG in PA embryos.

### The expression pattern of TET1, TET2, and TET3

**Figure 2:**
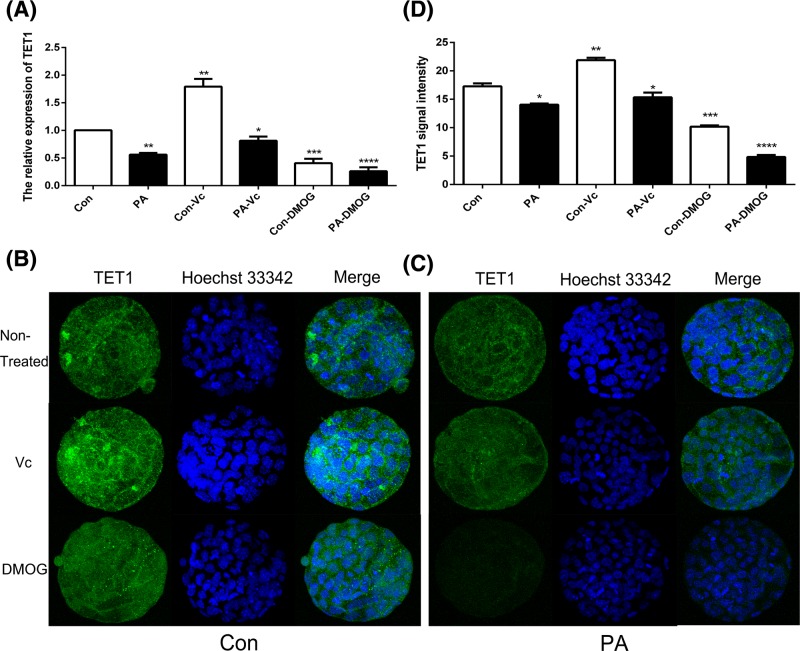
The expression of TET1 in Con and PA embryos The relative expression levels of TET1 was analyzed by qRT-PCR (**A**). IF localization of TET1 in Con (**B**) and PA (**C**) embryos. TET1 signal intensities were measured at blastula stage (**D**). The data are represented as the mean ± S.E.M. (*n*=3). *P<0.05, **P<0.01,*** *P*<0.005 and ****P<0.001 indicate statistically significant differences.

**Figure 3:**
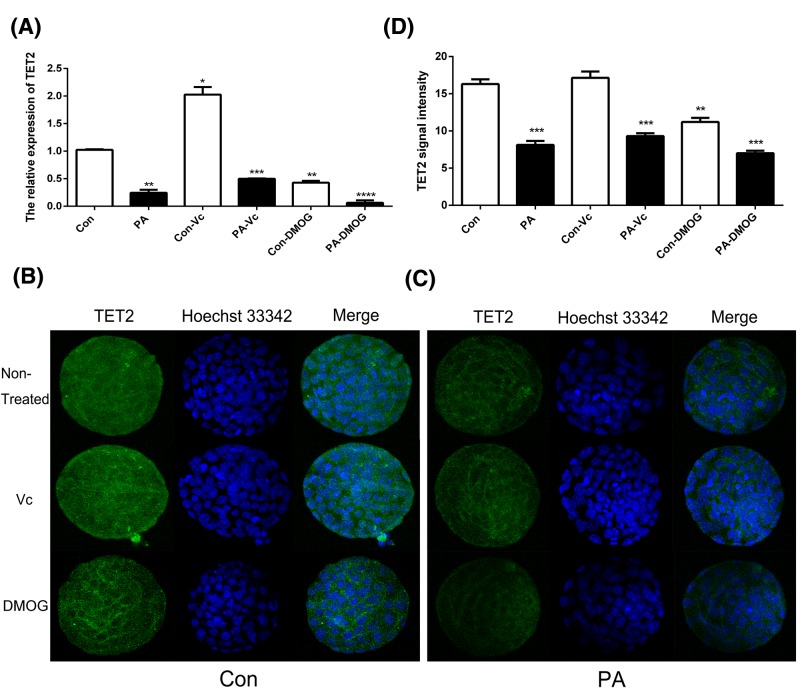
The expression of TET2 in Con and PA embryos The relative expression levels of TET2 were analyzed by qRT-PCR (**A**). IF localization of TET2 in Con (**B**) and PA (**C**) embryos. TET2 signal intensities were measured at blastula stage (**D**). The data are represented as the mean ± S.E.M. (*n*=3). *P<0.05, **P<0.01, ***P<0.005, and ****P<0.001 indicate statistically significant differences.

**Figure 4:**
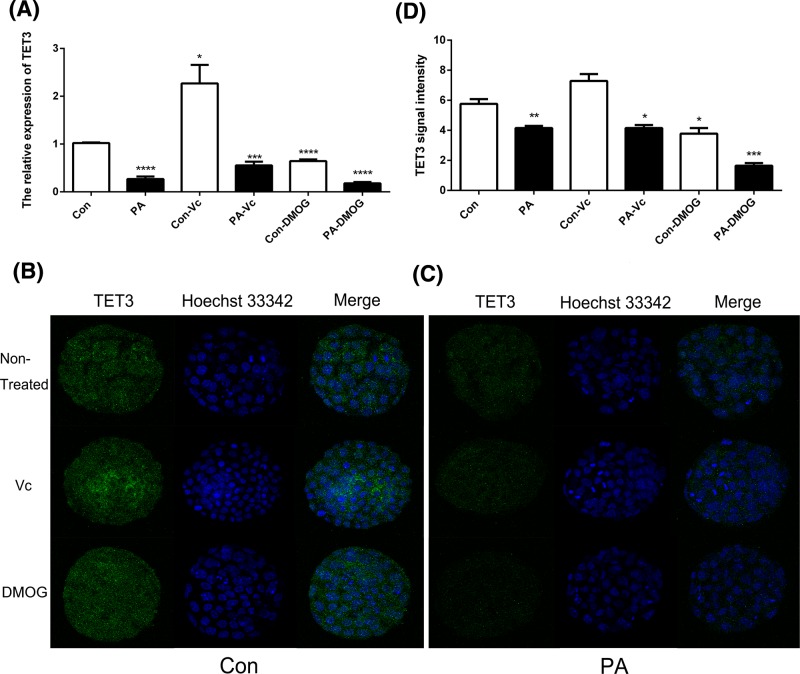
The expression of TET3 in Con and PA embryos The relative expression levels of TET3 were analyzed by qRT-PCR (**A**). IF localization of TET3 in Con (**B**) and PA (**C**) embryos. TET3 signal intensities were measured at blastula stage (**D**). The data are represented as the mean ± S.E.M. (*n*=3). *P<0.05, **P<0.01, ***P<0.005, and ****P<0.001 indicate statistically significant differences.

To investigate whether TET proteins were associated with PA embryonic development, the expression pattern of TET1, TET2, and TET3 was analyzed in the PA blastocyst stage. qPCR and IF staining results revealed that the expression of TET was significantly lower in the PA blastocyst stage compared with Con. As shown, TET1 (Figure 2), TET2 (Figure 3), and TET3 (Figure 4) expressions were stimulated by Vc and suppressed by DMOG. These results suggested that the expression of TET1, TET2, and TET3 was disrupted in PA embryos; moreover, Vc and DMOG regulated the expression pattern of TET proteins.

## Discussion

Genomic imprinting suffers from imprinting erasure and the establishment of epigenetic reprogramming in early mammalian development [12]. Although PA embryos contain exclusively maternal genomes, the PA model has been widely used to study biological research, especially for the investigation of fertilization and the imprinting process [13]. Compared with normal fertilized embryos, PA embryos can develop to the blastocyst stage as far as gestational day [14]. However, a low blastocyst rate of embryos has been observed in mice [15], porcine [16], and bovine PA embryos [17]. In this study, we addressed the question of whether PA development results in the abnormal expression of TET proteins, thus affecting blastocyst formation.

Treatment with Vc enhances TET expression [9,18,19]. Moreover, Vc improves embryo development, including the development of PA embryos, which might be associated with increased TET expression [20,21]. DMOG inhibits TET expression and negatively affects blastocyst formation in bovine PA embryo development [10,22]. To investigate the role of the TET proteins in PA embryo development, Vc and DMOG were used. Our results demonstrated that Vc improves the blastocyst rates of mouse PA embryos, whereas DMOG results in a decline in blastocyst formation. To further understand if TET affects blastocyst formation by PA embryos, TET1, TET2, and TET3 expressions were analyzed after Vc or DMOG treatment. TET1, TET2, and TET3 were necessary to induce the formation of 5hmC from 5mC in embryo development [23]. Knockdown of TET1 or TET2 reduces ESC proliferation but does not affect pluripotency in mice [24,25]. In addition, depletion of TET3, which is specifically enriched in the male pronucleus during embryonic development, results in an increased incidence of developmental failure in mice [4]. Our qPCR and IF staining results showed low expression of TET in PA embryos, in accordance with previous data [8]. Due to the lack of the paternal genome in PA embryos, loss expression of TET3 was observed in the PA embryos. When cultured with Vc, increased expression of TET proteins was observed. By contrast, DMOG suppressed TET expression in PA embryos. Thus, these results indicate that the expression of TETs, especially TET3, has an important role in PA embryonic development.

5hmC is specifically enriched in the sperm-derived chromosomes during preimplantation development [26]. Thus, the 5hmC level was gradually reduced in the PA embryos, in accordance with previous results [26–28]. Our results showed that Vc increased 5hmC levels in association with overexpression of TETs in mouse PA embryos. DMOG reduced 5hmC levels due to the loss of TET expression. The above results, together with previous studies, support a model in which TET3-driven hydroxylation targetting *de novo* methylation plays a key role in PA embryo development [29].

## Conclusion

In conclusion, the results demonstrated that the expression of TET proteins, especially TET3, and 5hmC levels were disrupted in PA embryos. Furthermore, the TET proteins regulated 5hmC and affected blastocyst formation following Vc and DMOG treatment. Taken together, the findings of the present study suggest that up-regulated expression of TET proteins improves PA embryo development by increasing 5hmC levels.

## Supporting information

**Table S1:** Primers for qRT-PCR analysis

## Abbreviations

DMOG: dimethyloxallyl glycine
ESC: Embryonic stem cell
hCG: human chorionic gonadotropin
IF: immunofluorescence
PA: parthenogenetic
PMSG: pregnant mare serum gonadotropin
PVA: Poly (vinyl alcohol)
qRT-PCR: quantitative real-time PCR
RT: room temperature
TET: Ten-Eleven translocation
Vc: vitamin C
5mC: 5-methylcytosine
5hmC: 5-hydroxymethylcytosine
6-DMAP: 6-(Dimethylamino) purine
α-KGDD: α-ketoglutarate- and Fe^2+^-dependent dioxygenase
