# Supplementary material for: Ascorbic acid improves parthenogenetic embryo development through TET proteins in mice
Source: Biosci Rep. 2019 Jan 11;39(1):BSR20181730. doi: 10.1042/BSR20181730 (PMC6328890; doi:10.1042/BSR20181730)
Supplement: Supplementary file 1 [file bsr20181730_Supp1.pdf]

Table S1 Primers for qRT-PCR analysis

Genes	Annealing (°C)	Primer sequences (5'→3')	Reference/accession
TET1	60	F: AGCTGGATTGAAGGAACAGG<br>R: GTCTCCATGAGCTCCCTGAC	[9]
TET2	60	F: AGAGCCTCAAGCAACCAAAA<br>R: ACATCCCTGAGAGCTCTTGC	[9]
TET3	60	F: TGCGATTGTGTCGAACAAATAGT<br>R: TCCATACCGATCCTCCATGAG	[9]
HPRT	60	F: CAGTACAGCCCCAAAATGGT<br>R: CAAGGGCATATCCAACAACA	[9]
